# Effects of a peer advocacy intervention on cervical cancer screening among social network members: results of a randomized controlled trial in Uganda

**DOI:** 10.1007/s10865-023-00418-6

**Published:** 2023-09-13

**Authors:** Glenn J. Wagner, Joseph K. B. Matovu, Margrethe Juncker, Eve Namisango, Kathryn Bouskill, Sylvia Nakami, Jolly Beyeza-Kashesya, Emmanuel Luyirika, Laura M. Bogart, Harold D. Green, Rhoda K. Wanyenze

**Affiliations:** 1 RAND Corporation, 1776 Main Street, Santa Monica, CA 90407 USA; 2 School of Public Health, Makerere University, Kampala, Uganda; 3 Faculty of Health Sciences, Busitema University, Mbale, Uganda; 4 Rays of Hope Hospice Jinja, Jinja, Uganda; 5 African Palliative Care Association, Kampala, Uganda; 6 Mulago Specialized Women and Neonatal Hospital, Kampala, Uganda; 7 School of Medicine, Makerere University, Kampala, Uganda; 8 University of Indiana Bloomington School of Public Health, Bloomington, IN USA

**Keywords:** Cervical cancer, Prevention, Peer advocacy, Intervention, Social network, Stigma, Uganda

## Abstract

Cervical cancer (CC) is the most common cancer among women in Uganda, yet lifetime CC screening is as low as 5%. Training women who have screened for CC to engage in peer advocacy could increase uptake of CC screening in social networks. We conducted a randomized controlled trial of a peer-facilitated, manualized, 7-session group intervention to train women to engage in CC prevention advocacy. Forty women recently screened for CC (index participants) enrolled and were assigned to receive the intervention (n = 20) or wait-list control (n = 20). Each index was asked to recruit up to three female social network members (alters) who had not been screened for CC (n = 103 enrolled alters). All index and alter participants were assessed at baseline and month-6 follow-up. All but one (n = 39; 98%) index and 98 (95%) alter participants completed the month 6 assessment. In multivariate regression models controlling for baseline outcome measures and demographic covariates, intervention alters were more likely to have been screened for CC at month 6 [67% vs. 16%; adjusted OR (95% CI) = 12.13 (4.07, 36.16)], compared to control alters. Data also revealed significant increased engagement in CC prevention advocacy, among both index and alter participants in the intervention group at month 6, compared to the control group. The intervention was highly effective in increasing CC screening uptake among social network members, and engagement in CC prevention advocacy among not only intervention recipients, but also targets of advocacy, suggesting the potential for wide dissemination of CC knowledge.

**Trial Registration.** NIH Clinical Trial Registry NCT04960748 (clinicaltrials.gov).

## Introduction

Cervical cancer (CC) is the most common cancer and accounts for ~ 25% of all cancer related deaths among women in Uganda, who have one of the highest incidence rates in the world at 54.8 per 100,000 (African Cancer Registry Network, 2018; Ferlay et al., 2018; International Agency of Cancer Registries, 2018). CC screening via visual inspection of the cervix with acetic acid (VIA), and thermal therapy for pre-cancerous lesions, are available for free or a low cost in some areas of Uganda. In contrast, radiotherapy is prescribed for advanced disease but scarcely available and too costly for most women. This highlights the importance of timely and periodic screening to prevent onset of cancerous lesions, yet it is estimated that as few as 5% of Ugandan women have ever screened for CC (Bruni et al., 2019; Nakisige et al., 2017; Ndejjo et al., 2016), and most (80%) have advanced disease (stage III or higher) when initiating care (Nakisige et al., 2017). WHO and Ugandan policy recommends CC screening every three years, which has evidence for preventing CC and promoting early detection of pre-cancerous lesions (Union for International Cancer Control, 2022; World Health Organization, 2019).

Barriers to CC screening include factors at the structural (poor access; few trained providers; national policies that frame CC as an outcome of the sexually transmitted human papillomavirus, which may stigmatize CC screening) (Dutta, Meyerson, et al., 2018; Nakisige et al., 2017), individual (younger age; low socioeconomic status; poor CC knowledge and awareness) (Nakisige et al., 2017; Ndejjo et al., 2017; Wanyenze et al., 2017), and interpersonal (exposure to intimate partner violence; stigma associated with fears and misconceptions regarding CC screening and treatment procedures) (Dutta, Haderxhanaj, et al., 2018; Ndejjo et al., 2017) levels. One approach to addressing the individual-level barriers to increased CC screening uptake is to empower women who have ever been screened for CC to act as advocates and encourage other women to get screened. Peer advocacy interventions have been effective at increasing prevention behaviors and reducing stigma in the context of HIV (Friedman et al., 2004; Latkin et al., 2003; Sikkema et al., 2000; Wagner et al., 2022), but we are unaware of any interventions designed to leverage and diffuse information through social networks to improve uptake of CC screening– this despite facilitators of CC screening including encouragement from others to get screened, and knowing someone who has screened for or been diagnosed with CC (Black et al., 2019).

**Fig. 1:**
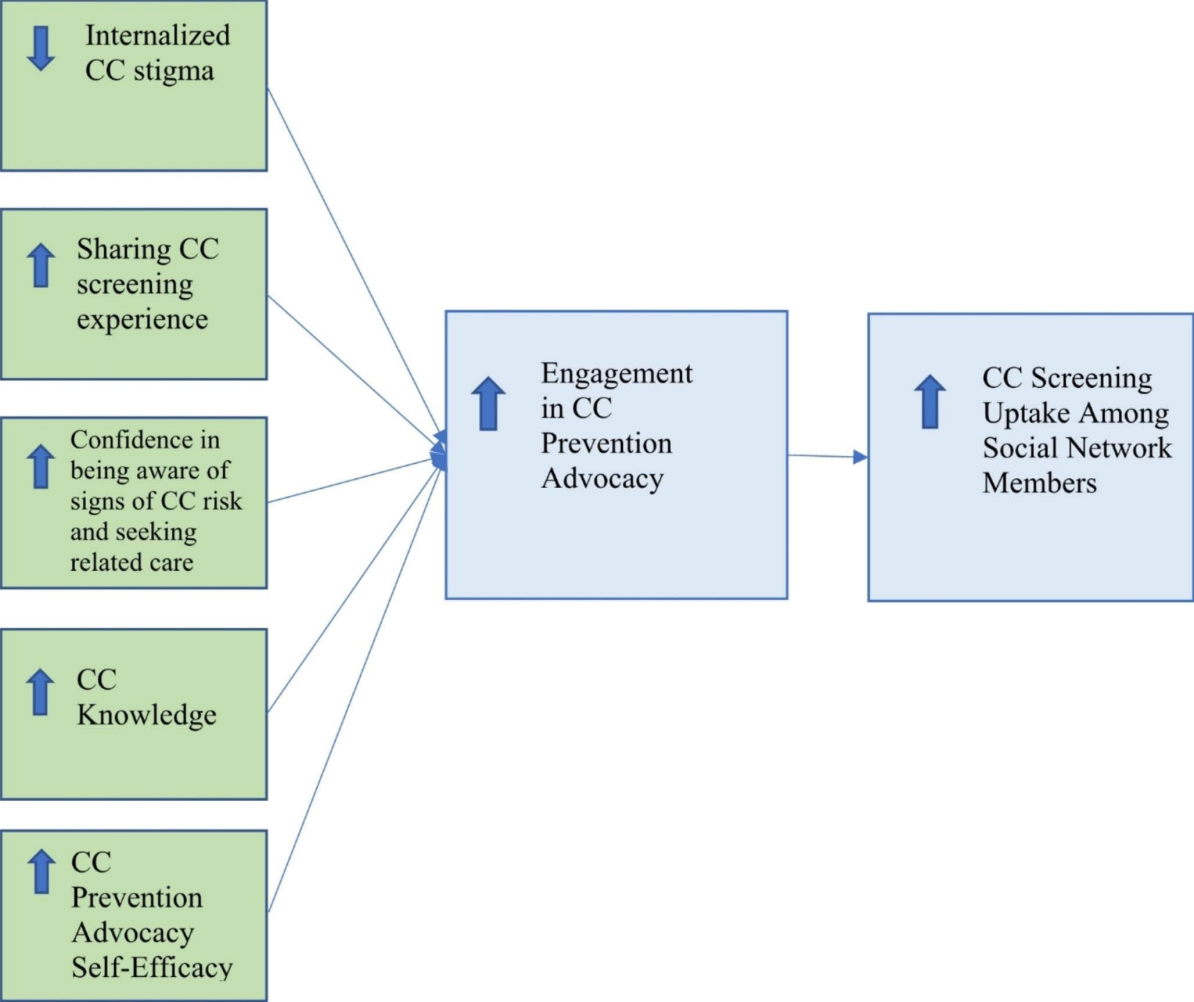
Conceptual framework for promotion of cervical cancer (CC) prevention advocacy among screened women to affect CC screening among social network members

Building on theories of social diffusion (Rogers, 1983), cognitive consistency (Festinger, 1957), and social influence (Broadhead et al., 1998), which posit that behavior change can be initiated by a few and diffused to others through modeling, advocacy, and shifts in social norms, we developed a social network-based advocacy group intervention to promote CC screening. As depicted in Fig. 1, the intervention seeks to empower and mobilize women who have ever been screened for CC to act as change agents for CC screening within their social networks by directly targeting stigma reduction, sharing of CC screening experience, knowledge of CC facts and myths, CC risk management, and advocacy skills building. The intervention was adapted from a similar intervention that we developed for use with people living with HIV to promote HIV prevention (Bogart et al., 2020).

We conducted a pilot randomized controlled trial of the intervention in which we enrolled women who had recently screened for CC to receive the intervention, as well as unscreened women from within their social networks (referred to as “alters”) to evaluate the effects of the intervention on alter uptake of CC screening. We hypothesized that the intervention would be associated with greater engagement in CC screening advocacy among intervention recipients, and greater uptake of CC screening among their social network members.

## Methods

**Study Design.** The study was a pilot randomized controlled trial of the multi-session, group advocacy training intervention for women who had recently screened for CC (referred to as index participants), with individual randomization on a 1:1 ratio to either the intervention or wait-list control groups (the latter of which received the intervention after data collection was completed). Randomization was stratified by age (under and over age 35) and history of CC-related treatment; a list of random group assignment codes for each strata was computer-generated. Participants were not blind to assignment; only the data analyst was blinded. Each index participant was asked to enroll up to three alters in their social network who had not screened for CC in the past 3 years. All participants (index and alter) were administered assessments at baseline and month 6, and received 30,000 Uganda shillings (~$8 USD) for each completed assessment. The primary outcome was alter CC screening over the 6-month follow-up period; secondary outcome was index participant reported engagement in CC screening advocacy. The study protocol was approved by the Makerere University School of Public Health Research and Ethics Committee, and cleared by the Uganda National Council for Science and Technology. The trial is registered with the NIH clinical trial registry (clinicaltrials.gov) and assigned the number NCT04960748 (registration date: 6/25/2021). Further details of the study protocol are available in a prior publication (Wanyenze et al., 2022).

**Study Setting**. The study took place in Namayingo, a rural district in the Busoga region of Uganda, and more specifically at Buyinja Health Center IV and Banda Health Center III, as these were the two health centers where CC screening and thermal therapy were available in the study setting. Women in this district could also be screened for CC through Rays of Hope Hospice Jinja (RHHJ), which conducts periodic mobile CC screening and thermal therapy “day camps”. Women who need biopsies are referred to Jinja Regional Referral Hospital (approximately 90 km from Namayingo), and if cancerous lesions are present, they are referred to the Uganda Cancer Institute, the leading and only tertiary public cancer care center located in Kampala. Women screened by RHHJ are registered in a database used to track them and facilitate further follow-up and outreach.

**Participants.** Recruitment was conducted in September, 2021. Women were eligible to enroll as index participants if they were age 18 years or older, had screened for CC within the past year (regardless of the result of the screening for cervical cancer risk), had stable health status (i.e., not in end stages of disease if CC was detected), and had shared their CC screening experience with at least one alter who was perceived to not have screened for CC in the past three years. Alter participants were eligible if they were at least 18 years of age, recruited by an enrolled index participant, and reported not being screened for CC in the past three years; these alters were women who had close relations with the recruiting index participant, given the frequency of their contact with the index and the fact that the index had shared their CC screening experience with the alter, as described in detail below.

Index participants were recruited through the RHHJ database of women who had received CC screening, and referrals from Buyinja and Banda health center providers. Recruitment of index participants was purposive in order to recruit a balance of women who screened positive for signs of CC risk (pre-cancerous or cancerous lesions), and women who screened negative, so that we could assess whether this factor was associated with the outcomes measure of engagement in CC prevention advocacy. An RHHJ staff member or health center provider informed eligible women of the study and those who expressed interest were referred to the study coordinator for formal eligibility screening and consent procedures. After providing written informed consent, women were administered the baseline assessment and then randomly assigned to the intervention or control arm. To recruit alters, data collected from the index baseline survey assessment of female social network members whom they had frequent contact with (up to 12 social network members per index participant, as described below) was used to randomly select five alters who knew the participant’s CC screening experience (or as many as there were if less than five). The index participant was asked if she was comfortable asking three of these alters to participate, and if so, was asked to call each selected alter at the end of the interview to describe the study in the presence of the coordinator, who scheduled a study visit for alters who expressed interest in participating. If an alter refused or could not be reached, a replacement was randomly selected from the list of alters who knew the index participant’s CC screening experience and whom the index participant was comfortable recruiting. The informed consent process for both index and alter participants was conducted in the local language preferred by the participant (Samia or Lusoga), by the study coordinator who was Ugandan and fluent in these local languages. The consent forms had been translated into the local languages using standard translation and back translation methods.

### Intervention

Following formative work that included six focus group discussions, three among women who had been screened for CC and three among women who had never been screened, we adapted the intervention manual to include aspects of stigma (primarily related to CC stemming from a sexually transmitted infection and that a positive screen may signify promiscuity, but also related to physical manifestations of disease), and misconceptions related to the screening procedure and possible side effects from screening and thermal therapy. Further details of this formative work are available elsewhere (Bouskill et al., Under review.) The intervention consisted of seven weekly group sessions. ***Session 1*** focused on addressing fears and concerns related to CC risk and use of self-compassion and peer support to overcome fears and internalized stigma, as well as introducing the overall vision for empowering women to become change agents for CC prevention and treatment. ***Session 2*** focused on building skills and decision making for sharing one’s personal CC screening experience, knowing to whom to disclose and when, and how to initiate and navigate disclosure and conversations about CC. ***Session 3*** built skills and motivation for recognizing signs of CC risk and seeking health services, so that the advocate’s own behavior was consistent with the behavior they encouraged in others, as well as instruction on facts and myths related to CC to facilitate accurate CC screening advocacy. ***Session 4*** introduced the concept of a social network and how one’s network can serve as a tool for CC prevention advocacy and dissemination of CC-related information. ***Sessions 5 and 6*** focused on the skills needed for successful CC prevention advocacy, including strategies for how to start and sustain conversations about CC, and effective communication skills (e.g., reflective listening, paraphrasing, open-ended questions). ***Session 7*** focused on peer solidarity and support to inspire a commitment to ongoing CC advocacy. The sessions were administered in a group format to facilitate the use of: sharing of experiences to build support, solidarity and motivation among participants; group problem solving and role playing to build skills and self-efficacy; setting personal goals regarding disclosure and advocacy; and take home activities to reinforce practice of new skills and generate personal experiences to be processed in the sessions. Each session lasted 120–150 min. Participants received 30,000 Uganda shillings (~$8 USD) for attending each session to cover transport costs.

The sessions were conducted using a structured facilitator manual, in the predominant local languages of Samia and Lusoga, by two peer facilitators from Namayingo who themselves had been screened for CC. The facilitators were trained by the senior investigators over three days. The supervisor of the facilitators observed the implementation of each session and provided feedback and further training as needed during weekly supervision.

### Measures

Assessments included a standard survey (index and alter participants) and social network assessment (index participant only), which were administered in either Samia or Lusoga (depending on the preference of the participant) using Network Canvas computer assisted software. Each measure was assessed with both index and alter participants, unless otherwise noted. Measures were translated using standard translation/backtranslation methodology. CC screening and treatment utilization were verified with abstracted medical chart data. All measures were developed by the study team, except those in which an attribution is cited. For measures developed by the study team that included at least three items, we cite internal reliability statistics (Cronbach’s alpha).

**Social network assessment**. Each index participant was asked to list up to 12 women in their social network (referred to as “alters”) with whom they interacted most. For each alter, we gathered information to assess network composition (e.g., age, HIV status, relation to and frequency of contact with index participant; level of trust in the alter; perceived history with CC screening and treatment; knowledge of index’s CC screening and treatment). Our prior research shows that alter health seeking behaviors can be accurately reported by index participants (Green Jr, et al., 2014).

***CC screening and treatment***. Data were collected to determine if the participant had ever been screened for CC using visual inspection using acetic acid (VIA) or pap smear, and if so, when. For participants who had been screened, it was determined if the screening resulted in pre-cancerous lesions or potential cancerous lesions, in separate items; if either type of lesion was reported, receipt of corresponding procedure or treatment (cryotherapy or thermal therapy for pre-cancerous; biopsy to confirm diagnosis, and radiation, chemotherapy or surgery treatment for cancerous) was assessed.

***CC prevention advocacy*** was assessed with six items in which respondents reported the frequency of discussing CC-related topics (e.g., importance of CC screening, how and where to get screened, importance of getting treatment if signs of CC risk are present) with women they know in the past six months. Response options ranged from 1 ‘not at all’ to 5 ‘very much’; mean item scores were calculated and higher scores reflected greater engagement in advocacy (Cronbach’s alpha = 0.95).

***CC screening advocacy*** conducted by index participants with specific alters was assessed from the perspective of the index participant (for all alters named in the social network assessment), and the alter participants enrolled in the study (as part of their survey assessment). In the social network assessment, for each alter named, the index participant was asked if they had (1) talked with the alter about the importance of CC screening, (2) encouraged the alter to get screened, (3) provided information about where and how to get screened, and (4) provided direct support to the alter to get screened (e.g., taking them to the clinic); the response option for each of these four responses was 0 ‘no’ or 1 ‘yes’, and the mean item score was calculated for each alter as well as across all alters named by the index participant. In the alter survey, respondents were asked if the index participant had discussed, encouraged, provided information, and provided direct support to facilitate the alter getting screened for CC; mean item score was calculated for each alter interviewed, as well as across all participating alters recruited by the index participant.

***Internalized CC stigma*** was assessed among index participants, using 5 items adapted from a scale of HIV internalized stigma (e.g., My cervical cancer screening makes me feel ashamed of myself) (Kalichman et al., 2009). Mean item score was calculated and higher scores reflect greater stigma; Cronbach’s alpha = 0.57.

***CC enacted stigma*** was assessed among alter participants with six items adapted from measures developed by Marlow & Wardle (Marlow & Wardle, 2014) and Cho et al. (Cho et al., 2013). Participants were asked to rate their agreement with statements (e.g., A woman with cervical cancer is to blame for her condition; I feel uncomfortable when I am around women with cervical cancer) by indicating they 1 ‘disagree’, 2 ‘I neither agree nor disagree. I do not have a feeling either way’ or 3 ‘agree.’ Mean item score was calculated and higher scores reflect greater stigma; Cronbach’s alpha = 0.40.

***Sharing of CC screening experience*** was assessed for index participants, by asking respondents to what extent they had shared their CC screening result with sexual partners, family, and friends, in separate questions; higher mean item score reflects greater disclosure (Cronbach’s alpha = 0.74).

***CC knowledge*** was assessed with 16 statements or questions reflecting the etiology, prevention and treatment of CC; a sum of correct responses was calculated (Cronbach’s alpha = 0.75).

***CC health services utilization self-efficacy*** was assessed with three items that measured confidence to notice a symptom of CC risk, seek health services for a symptom of CC risk, and obtain treatment if screening revealed signs of CC risk; higher mean item score reflects greater self-efficacy (Cronbach’s alpha = 0.64).

***CC prevention advocacy self-efficacy*** was assessed with three items assessing confidence to start a conversation about the need for: CC screening, treatment for signs of CC risk, and telling someone about their CC screening experience; higher mean item score reflects greater self-efficacy (Cronbach’s alpha = 0.85).

***Demographic and background characteristics*** included age, level of formal education completed, relationship status and HIV status.

### Data analysis

Descriptive and bivariate (2-tailed independent t-tests; chi square tests) statistics were used to compare baseline sample characteristics of index and alter participants in the control versus intervention arms, in separate analyses. To examine intervention effects on index participant engagement in CC prevention advocacy, and alter participant uptake of CC screening, as well as other index and alter outcomes, we conducted multiple linear regression analysis for continuous outcomes (e.g., CC prevention advocacy) and multiple logistic regression analysis for CC screening. In each model, the month 6 measure of the outcome was the dependent variable, while independent variables included the baseline measure of the dependent variable (except for the outcome of alter CC screening, since all alter participants had never been screened for CC prior to enrollment), and an indicator of study arm, as well as covariates (age < 36 years, any secondary education, presence of a main sex partner, HIV status). If a measure was missing at month 6, the baseline measure of the variable was used to replace the missing value; for CC screening, missing data at month 6 was classified as not screened, which is similar to an intent-to-treat approach. All regression models involving alter data controlled for clustering at the level of each index (i.e., all the alters recruited by a specific index, as well as the index participant themselves, represent a single cluster) by using a SurveyReg or SurveyLogistic routine in SAS 9.2.

## Results

### Sample characteristics

Forty women (20 who receive their healthcare from Buyinja Health Center IV, 20 from Banda Health Center III) who had screened for CC within the past year, as identified from the RHHJ database or provider referral, were screened for eligibility– all of whom were eligible and decided to enroll in the study as index participants (i.e., none refused enrollment); 20 were randomly assigned to the intervention group and 20 to the wait-list control group. Twenty-four (60%) had screened positive for signs of pre-cancerous lesions, and each received treatment (17 received thermal therapy, 7 received cryotherapy), while the remaining 16 screened negative for any sign of CC risk. From these 40 index participants, 103 alters were recruited to enroll in the study (58 by intervention index participants, 45 by control index participants), all of whom reported never being screened for CC.

Table 1 shows the characteristics of the index and alter participants, by study arm. Most index participants were age 36 years or older (58%), compared to just 38% of alter participants, while both index and alter participants predominantly had a main sexual partner (> 80%), did not have any secondary education (> 70%), and were not HIV-positive (> 90%). The index participants in the intervention arm did not differ from those in the control group on any of these background characteristics, while alter participants in the intervention arm were more likely to have any secondary education (39.7% vs. 15.6%, p = .01) and have a main sex partner (89.7% vs. 71.1%, p = .02) compared to those in the control group. The month 6 assessment was completed by 39 (97.5%) index participants and 98 (95.1%) alters.

**Table 1:** Baseline sample characteristics by arm, among index (n = 40) and alter (n = 103) participants

	Index Participants [n (%)]				Alter Participants [n (%)]			
	Total (n = 40)	Control (n = 20)	Intervention (n = 20)	p/FET	Total (n = 103)	Control (n = 45)	Intervention (n = 58)	p/FET
Age > 35 years	23 (57.5%)	11 (55.0%)	12 (60.0%)	0.75	39 (37.9%)	19 (42.2%)	20 (34.5%)	0.42
Any secondary education	9 (22.5%)	3 (15.0%)	6 (30.0%)	0.45	30 (29.1%)	**7 (15.6%)**	**23 (39.7%)**	**0.01**
Has a main partner	35 (87.5%)	16 (80.0%)	19 (95.0%)	0.34	84 (81.6%)	**32 (71.1%)**	**52 (89.7%)**	**0.02**
HIV-positive	3 (7.5%)	2 (10.0%)	1 (5.0%)	1.00	6 (5.8%)	4 (8.9%)	2 (3.4%)	0.40
Has children	37 (92.5%)	18 (90.0%)	19 (95.0%)	1.00	98 (95.1%)	41 (91.1%)	57 (98.3%)	0.17

FET = Fisher’s Exact Test

The 20 index participants in the intervention arm were divided into two groups of ten to receive the 7-session intervention; 19 (95%) attended all seven sessions.

### Intervention effects on cervical cancer prevention advocacy and related outcomes among index participants at month 6

In multivariate linear regression models controlling for baseline measures of the dependent variable and covariates, intervention index participants reported greater CC prevention advocacy at month 6, along with greater advocacy for CC screening among named alters, compared to index participants in the control group; alters recruited by intervention index participants also reported greater receipt of CC screening advocacy from the index, compared to those of control index participants (see Table 2). Index participants in the intervention arm also reported greater levels of sharing of CC screening experience, CC knowledge, CC prevention advocacy self-efficacy, and CC health service utilization self-efficacy at month 6, compared to index participants in the control group (see Table 2). The two groups did not differ with respect to CC internalized stigma or the proportion of named alters who knew the index participant’s CC screening result.

**Table 2:** Intervention effects on index participant engagement in cervical cancer (CC) prevention advocacy, and related outcomes, at month 6, controlling for baseline levels of the outcome and background characteristics

Outcome	Baseline			Month 6			
	Control (n = 20)	Intervention (n = 20)	p	Control (n = 19)	Intervention (n = 20)	p	Beta (SE); p*
Sharing of CC screening result with others	1.47 (0.62)	1.62 (0.39)	0.37	**1.58 (0.48)**	**1.93 (0.21)**	**0.006**	**0.22 (0.10); 0.045**
% alters who know the index participant’s CC screening result	91.5% (23.1)	87.8% (24.5)	0.62	94.2% (18.2)	100% (0)	0.17	**0.06 (0.03); 0.09**
CC knowledge	8.55 (2.80)	10.05 (3.36)	0.13	**8.50 (2.37)**	**15.70 (0.66)**	**< 0.001**	**7.00 (0.60); <0.001**
CC internalized stigma	1.21 (0.35)	1.07 (0.16)	0.12	1.08 (0.29)	1.00 (0.00)	0.13	-0.01 (0.04); 0.77
CC health services utilization self-efficacy	8.70 (1.40)	8.85 (1.28)	0.73	**7.40 (2.16)**	**10.00 (0.00)**	**< 0.001**	**2.56 (0.50); <0.001**
CC prevention advocacy self-efficacy	9.75 (0.46)	9.72 (0.49)	0.83	**8.15 (1.98)**	**10.00 (0.00)**	**< 0.001**	**1.97 (0.45); <0.001**
CC prevention advocacy	3.23 (1.13)	3.57 (1.16)	0.35	**2.90 (1.10)**	**4.98 (0.11)**	**< 0.001**	**1.84 (0.22); <0.001**
CC screening advocacy across all alters (reported by index)	1.96 (0.19)	1.85 (0.51)	0.39	**2.03 (0.06)**	**2.19 (0.21)**	**0.004**	**0.17 (0.05); 0.001**
CC screening advocacy across all alters (reported by enrolled alters)	2.00 (0.00)	2.03 (0.10)	0.16	**1.97 (0.27)**	**2.26 (0.31)**	**0.006**	**0.33 (0.11); 0.005**

* Coefficient for main effect of the intervention, from linear regression model with the outcome measure at month 6 as the dependent variable, and independent variables being the baseline measure of the outcome, treatment condition (intervention or control), and background characteristic covariates (age, secondary education, whether index had a main sex partner, HIV status)

SE = standard error

### Intervention effects on cervical cancer screening, and other related outcomes, among alter participants at month 6

All enrolled alters reported never having been screened for CC at baseline. Conversely, at month 6, 38 (66.7%) of 57 intervention alters who had completed the assessment had been screened for CC, compared to 7 (17.1%) of 41 control alters; after classifying the five cases with missing data at month 6 as “not screened”, 66.5% (38/58) of the intervention alters had been screened, compared to 15.6% (7/45) of control alters. Among the 45 alters who were screened, 3 were found to have pre-cancerous lesions and received thermal therapy, and two other screenings suggested potential cancer (neither woman received treatment, but one did receive a biopsy).

Among the alters named by the index participants in their survey assessments (means of 7.8 and 8.9 alters named per index at baseline and month 6, respectively), no alters were perceived by the index to have been screened for CC at baseline; however, at month 6, 57.3% (SD = 23.7) of alters in the intervention group were perceived to have been screened for CC since baseline, compared to 14.4% in the control group [beta (SE) = 0.43 (0.06), p < .001]. Among enrolled alter participants, if the index participant who recruited the alter reported during the month 6 assessment that the alter had screened for CC, this perception was accurate for 88.2% of the cases.

In multivariate regression models controlling for baseline measures of the dependent variable (except for CC screening) and covariates, alter participants in the intervention group were much more likely to have been screened for CC at month 6 [adjusted OR (95% CI) = 12.13 (4.07, 36.16)], and a greater percentage of named alters were perceived to have been screened by intervention index participants at month 6 [adjusted beta (SE) = 0.40 (0.07); p < .001], compared to counterparts in the control group (see Table 3). Alter participants in the intervention arm also reported greater engagement in CC prevention advocacy, CC knowledge, CC health services utilization self-efficacy, and receipt of CC screening advocacy from the index participant at month 6, compared to alters in the control group (see Table 3); CC enacted stigma did not differ between the two groups of alters.

**Table 3:** Intervention effects on alter uptake of cervical cancer (CC) screening, and related outcomes, at month 6, controlling for baseline levels of the outcome and background characteristics

Outcome	Baseline			Month 6			
	Control (n = 45)	Intervention (n = 58)	p	Control (n = 41)	Intervention (n = 57)	p	OR (95% CI); or Beta (SE); p*
Screened for CC since baseline	--	--	--	**15.6%**	**63.8%**	**< 0.001**	**12.13 (3.18, 46.29)**
% alters perceived to be CC screened (index)	0	0		**14.4% (13.8)**	**57.3% (23.7)**	**< 0.001**	**0.40 (0.07); <0.001**
CC knowledge	4.76 (2.60)	5.57 (2.83)	0.14	**6.13 (2.93)**	**11.48 (3.60)**	**< 0.001**	**4.80 (0.60); <0.0001**
CC enacted stigma	1.76 (0.57)	1.81 (0.58)	0.63	1.60 (0.36)	1.65 (0.41)	0.55	0.07 (0.07); 0.33
CC health services utilization self-efficacy	6.96 (1.57)	7.40 (1.46)	0.14	**6.59 (2.11)**	**9.47 (4.20)**	**< 0.001**	**2.75 (0.72); 0.0005**
CC prevention advocacy	1.76 (0.48)	1.83 (0.68)	0.55	**1.69 (0.69)**	**3.41 (1.36)**	**< 0.001**	**1.75 (0.27); <0.0001**
Receipt of CC screening advocacy from index	2.00 (0.00)	2.03 (0.18)	0.16	**1.98 (0.50)**	**2.26 (0.44)**	**0.004**	**0.32 (0.11); 0.007**

* Odds ratio (OR) [and 95% confidence interval (CI)] or beta coefficient [and standard error (SE)] for main effect of the intervention, from logistic or linear regression model with the outcome measure at month 6 as the dependent variable, and independent variables being the baseline measure of the outcome (except for CC screening uptake, since all alters had never screened at baseline), treatment condition (intervention or control), and background characteristic covariates (age, secondary education, whether alter had a main sex partner, HIV status). The model for percent of alters perceived to have been screened for CC did not include alter background characteristic covariates, since this outcome was reported by index participants

## Discussion

In what may be the first study of a social-network based, peer-led group intervention to empower women who had been screened for cervical cancer (CC) to engage in CC prevention advocacy, our findings showed that the intervention was feasible and highly acceptable with near perfect attendance to the multi-session intervention. Effects were promising, as not only did the intervention increase CC prevention and screening advocacy among index participants, but also dramatically increased CC screening uptake among their social network members. The study findings also provided empirical validation of the intervention’s conceptual framework, with significant changes in most processes directly targeted by the intervention components.

The primary goal of the intervention was to increase uptake of CC screening among women in the social networks of those participating in the intervention. Our findings showed a strong effect of the intervention on this outcome, as alters of the women in the intervention group had odds tenfold higher of getting screened for CC during the six-month follow-up period of the study, compared to alters in the control group. This effect was not only true of the alters that were enrolled in the study, for whom CC screening was validated through medical chart abstraction, but also the index participant’s larger network of women based on the self-report of the index participant. Low uptake of CC screening in Uganda is likely due to poor access to low cost screening services in much of the rural-dominant Ugandan population (Nakisige et al., 2017), in addition to other barriers that may be structural (e.g., national policies that frame CC as being an outcome of a sexually transmitted infection, which can stigmatize CC screening), individual (e.g., poor knowledge of CC and availability of CC screening and treatment) or interpersonal (e.g., intimate partner violence; stigma) (Dutta, Haderxhanaj, et al., 2018; Dutta, Meyerson, et al., 2018; Ndejjo et al., 2017; Wanyenze et al., 2017); however, our study provides evidence that if screening services are available, women will get screened with encouragement and information provided by women who they know and respect, and have experienced the screening procedure themselves. This evidence adds to the body of literature that suggests peer advocacy interventions can help to promote health services utilization (Friedman et al., 2004; Latkin et al., 2003; Sikkema et al., 2000; Wagner et al., 2022).

The most direct target of the intervention was engagement in CC prevention and screening advocacy on the part of the index participants—women who had direct experience with being screened for CC, and in many cases also treated for pre-cancerous lesions. The data showed a significant increase in CC prevention advocacy with social network members among index participants in the intervention group, as measured by the self-report of the index participants as well as the report of their recruited alters. Not only did the intervention result in increased CC prevention advocacy among index participants in the intervention group, but also increased advocacy conducted by alters recruited into the study by intervention index participants. This is particularly noteworthy, as it suggests a transfer of advocacy with the initial targets of advocacy increasing their own personal advocacy towards other women in their network. This transference of advocacy and awareness about the importance of CC screening has the potential to transform CC prevention throughout an entire community of women and highlights the potential power of network-based peer advocacy interventions.

The conceptual framework for the development of the intervention garnered empirical validation from the study data. The framework posits that low internalized CC stigma, sharing of CC screening experience, knowledge about the facts and myths related to CC, and self-efficacy with regards to being able to identify and seek out health services for signs of CC risk, as well as confidence in being able to engage in CC-related advocacy—are all key to setting the foundation for increased engagement in successful CC prevention advocacy (see Fig. 1). Accordingly, the content of intervention sessions specifically targeted these factors. Our data provide empirical support for this model, with the intervention resulting in significant longitudinal improvement in each of these constructs, with the exception of internalized CC stigma. Levels of internalized stigma were generally very low in the sample, creating a likely ceiling effect in terms of being able to detect significant stigma reduction.

There are several aspects of participant recruitment that may have contributed to selection bias and limiting of the generalizability of our findings. Index participants were women who had recently received CC screening services from their provider or RHHJ, and all agreed to enroll once they were informed of the study. This may be a reflection of their gratitude for CC-related health services and a level of motivation to participant that may limit the extent to which the sample is representative of the general population of women who have screened for CC. Index participants who enrolled in the study knew that they would be trained to engage in CC prevention advocacy, and thus were likely motivated to be advocates; this motivation may be associated with greater CC knowledge and other constructs we measured, and not representative of the general population of women who had recently screened for CC. The purposive recruitment of index participants such that a disproportionately higher proportion of the sample had screened positive for CC risk compared to the general population, also represents a bias. There was also a selection bias related to the alter participants, as index participants needed to be comfortable recruiting these alters; these alters may not be representative of all women in the social networks of the index participants. Furthermore, the presence of the transportation refund for attending intervention sessions and completing study assessments may have incentivized some participants to enroll and contributed to a selection bias.

Other study limitations included the fact that validated measures or measures used by other research groups were not available for most constructs, resulting in the need for our team to develop many of the measures used. Internal reliability statistics ranged from low to high across these measures, resulting in the need for further psychometric evaluation is future studies. We relied on index perception of alters, rather than direct reports from alters, for most alter characteristics, which renders such data vulnerable to inaccuracy and bias. However, for the primary outcome, alter CC screening, we were able to link data from index perception and chart abstraction and found that index perception was very accurate. Other limitations include the small sample size and limited statistical power, as well as lack of allocation concealment for index participants and study personnel, which could introduce bias and contribute to differences between the study arms on alter characteristics.

In conclusion, the findings from this randomized controlled trial of a multi-session group intervention that trains women who have been screened for CC to act as change agents for CC prevention by engaging in CC prevention advocacy among women in their social networks, showed strong effects on CC screening uptake among social network members. Social network members of women in the intervention group had over tenfold the odds of getting screened for CC in the 6 months following enrollment. The intervention also had strong effects on increased engagement in CC prevention and screening advocacy among index participants, as well as more sharing of CC screening experiences, and greater self-efficacy related to advocacy and CC health service utilization, as posited by the theoretical framework that guided the development of the intervention. CC prevention advocacy also increased among alter participants in the intervention group, suggesting that this peer advocacy intervention may have a potential ripple effect on advocacy, resulting in a network-based dissemination of CC information that could mobilize a whole network of women to get screened for CC. So long as structural barriers are limited and women are able to access affordable CC screening, this type of peer advocacy intervention could be a critical game changer for increasing demand for CC screening, and saving the lives of many women. A larger evaluation of the intervention is needed to establish whether these strong results can be replicated in multiple settings, and to better understand the transfer of advocacy mobilization and empowerment within social networks and communities of women at risk for CC.

## Data Availability

De-identified dataset and statistical code are available to researchers upon submission of proposal and review by the study team.

## References

[CR2] Black E, Hyslop F, Richmond R (2019). Barriers and facilitators to uptake of cervical cancer screening among women in Uganda: A systematic review. BMC women’s health.

[CR3] Bogart LM, Matovu JK, Wagner GJ, Green HD, Storholm ED, Klein DJ, Marsh T, MacCarthy S, Kambugu A (2020). A pilot test of game changers, a social network intervention to empower people with HIV to be prevention advocates in Uganda. AIDS and Behavior.

[CR4] Bouskill, K., Wagner, G., Gizaw, M., Matovu, J., Juncker, M., Namisango, E., Nakami, S., Beyeza-Kashesya, J., Luyirika, E., & Wanyenze, R. (Under review.). Understanding women’s and men’s perspectives on cervical cancer screening and stigma in Uganda. 10.1186/s12885-024-12671-2 PMC11293159 39090654

[CR5] Broadhead RS, Heckathorn DD, Weakliem DL, Anthony DL, Madray H, Mills RJ, Hughes J (1998). Harnessing peer networks as an instrument for AIDS prevention: Results from a peer-driven intervention. Public health reports.

[CR6] Bruni, L., Albero, G., Serrano, B., Mena, M., Gómez, D., Muñoz, J., & Bosch, F. X. (2019). & S, d. S. *Human Papillomavirus and Related Diseases in Uganda. Summary Report*

[CR7] Cho J, Choi E, Kim SY, Shin DW, Cho BL, Kim C, Koh DH, Guallar E, Bardwell WA, Park JH (2013). Association between cancer stigma and depression among cancer survivors: A nationwide survey in Korea. Psycho-Oncology.

[CR8] Dutta, T., Haderxhanaj, L., Agley, J., Jayawardene, W., & Meyerson, B. (2018a). Peer reviewed: association between individual and intimate partner factors and cervical cancer screening in Kenya. Preventing chronic disease, 15. 10.5888/pcd15.180182 PMC6307831 30576277

[CR9] Dutta T, Meyerson B, Agley J (2018). African cervical cancer prevention and control plans: A scoping review. Journal of cancer policy.

[CR10] Ferlay, J., Ervik, M., Lam, F., Colombet, M., Mery, L., Piñeros, M., Znaor, A., Soerjomataram, I., & Bray, F. (2018). Global cancer observatory: cancer today. *Lyon, France: international agency for research on cancer*, 1–6.

[CR11] Festinger, L. (1957). *A theory of cognitive dissonance* (2 vol.). Stanford university press.

[CR12] Friedman SR, Maslow C, Bolyard M, Sandoval M, Mateu-Gelabert P, Neaigus A (2004). Urging others to be healthy:“Intravention” by injection drug users as a community prevention goal. AIDS Education and Prevention.

[CR13] Green HD, Hoover MA, Wagner GJ, Ryan GW, Ssegujja E (2014). Measuring agreement between egos and alters: Understanding informant accuracy in personal network studies. Field Methods.

[CR15] Kalichman SC, Simbayi LC, Cloete A, Mthembu PP, Mkhonta RN, Ginindza T (2009). Measuring AIDS stigmas in people living with HIV/AIDS: The internalized AIDS-Related Stigma Scale. AIDS care.

[CR16] Latkin CA, Sherman S, Knowlton A (2003). HIV prevention among drug users: Outcome of a network-oriented peer outreach intervention. Health Psychology.

[CR17] Marlow LA, Wardle J (2014). Development of a scale to assess cancer stigma in the non-patient population. BMC cancer.

[CR18] Nakisige C, Schwartz M, Ndira AO (2017). Cervical cancer screening and treatment in Uganda. Gynecologic oncology reports.

[CR20] Ndejjo R, Mukama T, Musabyimana A, Musoke D (2016). Uptake of cervical cancer screening and associated factors among women in rural Uganda: A cross sectional study. PloS one.

[CR19] Ndejjo, R., Mukama, T., Kiguli, J., & Musoke, D. (2017). Knowledge, facilitators and barriers to cervical cancer screening among women in Uganda: A qualitative study. BMJ open, 7(6), e016282. 10.1136/bmjopen-2017-016282 PMC5541520 28606908

[CR21] Rogers, E. M. (1983). *Diffusion of innovations* (2nd edition ed.). Simon and Schuster.

[CR22] Sikkema KJ, Kelly JA, Winett RA, Solomon LJ, Cargill VA, Roffman RA, McAuliffe TL, Heckman TG, Anderson EA, Wagstaff DA (2000). Outcomes of a randomized community-level HIV prevention intervention for women living in 18 low-income housing developments. American journal of public health.

[CR24] Wagner GJ, Bogart LM, Klein DJ, Green HD, Nampiima J, Kambugu A, Matovu JKB (2022). Association of Condom Use Advocacy with Perceived Condom Use among Social Network Members: The mediating role of advocates’ internalized HIV stigma and own condom use. Aids And Behavior.

[CR25] Wanyenze RK, Bwanika JB, Beyeza-Kashesya J, Mugerwa S, Arinaitwe J, Matovu JK, Gwokyalya V, Kasozi D, Bukenya J, Makumbi F (2017). Uptake and correlates of cervical cancer screening among HIV-infected women attending HIV care in Uganda. Global health action.

[CR26] Wanyenze, R. K., Matovu, J. K. B., Bouskill, K., Juncker, M., Namisango, E., Nakami, S., Beyeza-Kashesya, J., Luyirika, E., & Wagner, G. J. (2022). Social network-based group intervention to promote uptake of cervical cancer screening in Uganda: Study protocol for a pilot randomized controlled trial. *Pilot Feasibility Studies*, *8*(1), 247. 10.1186/s40814-022-01211-z 10.1186/s40814-022-01211-z PMC9727870 36476609

[CR1] African Cancer Registry Network (2018). *Kampala Cancer Registry* Retrieved February 19 2018 from http://afcrn.org/membership/membership-list/81-kampala-uganda

[CR14] International Agency of Cancer Registries (2018). *Kampala Cancer Registry Profile Page*.

[CR23] Union for International Cancer Control (UICC) (2022). *Cervical cancer elimination in Africa: where are we now and where do we need to be?*.

[CR27] World Health Organization (2019). WHO guidelines for the use of thermal ablation for cervical pre-cancer lesions. 31661202

